# Early Childhood Predictors of Teen Dating Violence Involvement at Age 17

**DOI:** 10.1007/s10964-022-01664-8

**Published:** 2022-08-06

**Authors:** Noemí Pereda, Ana M. Greco, Diego A. Díaz-Faes, Manuel Eisner, Denis Ribeaud

**Affiliations:** 1 Research Group on Child and Adolescent Victimization (GReVIA), University of Barcelona, Barcelona, Spain; 2 Institute of Neurosciences (UBNeuro), University of Barcelona, Barcelona, Spain; 3 Open University of Catalonia, Barcelona, Spain; 4 Institute of Criminology/Violence Research Center, University of Cambridge, Cambridge, UK; 5 Jacobs Center for Productive Youth Development, University of Zurich, Zürich, Switzerland

**Keywords:** Dating violence, Predictors, Longitudinal, z-proso, Risk factors

## Abstract

The distal relationship between risk factors in childhood and subsequent dating violence in late adolescence has not often been explored using longitudinal data. This study aims to shed light on the problem of dating violence by examining children’s backgrounds at age 7 and the link to the future involvement in dating violence at age 17 using the first and seventh waves of the Zurich Project on the Social Development from Childhood to Adulthood (z-proso, *n* = 644). The sample consists of 644 multiethnic adolescents (57.14% female, *M* = 17.47, *SD* = 0.37), mainly Swiss-born (90%), though more than half of their parents (60%) were born in another country. A latent class analysis was applied to identify three different profiles (a) zero (or minimal) involvement in teen dating violence, (b) perpetrators/victims of controlling behaviors, and (c) perpetrators/victims of controlling behaviors and of physical violence. Participants who were corporally punished and/or victims of bullying at age 7 were significantly more likely to belong to the controlling and physical violence profile than children in the non-violent class. These results suggest a certain chronicity of the effects of violent experiences in early childhood on the patterns of romantic relationships at 17 years old.

## Introduction

Teen dating violence is a public health issue which involves the threat or actual use of physical, sexual, emotional violence, or combination of one or more, against the intimate partner within the context of early- and mid-adolescent romantic relationships (Exner-Cortens, 2014). It can be comprehensively understood as the result of the complex interplay of multiple factors cascading across the life course, yielding a plethora of risk and protective factors at individual, relationship, and social environmental levels which generate different interpersonal contexts for dating violence (Hébert et al., 2019). However, most adolescent dating violence studies have used cross-sectional designs (Foshee et al., 2004), and the few longitudinal studies available have addressed its correlates from late childhood or early adolescence. This study addresses this gap in our knowledge by focusing on the distal relationship between risk factors in childhood and subsequent dating violence in middle adolescence.

### The Prevalence of Teen Dating Violence

The extent of teen dating violence is still not well known. Studies from different cultures and contexts suggest that adolescents quite frequently experience violence in their dating relationships, and that this violence may be severe (Jennings et al., 2017). Regarding its epidemiology, a meta-analysis conducted in Western high-income countries reported an overall prevalence of 20% for physical violence (i.e., slapping, hitting, punching, kicking, beating, or throwing objects) and 9% for sexual violence (i.e., sexual activity forced on the partner ranging from unwanted touching to forced penetration) in dating relationships, which highlights the extent of the problem (Wincentak et al., 2017). However, the rates vary widely between studies, ranging from 1% to 61% for physical violence and from <1% to 54% for sexual violence. Controlling behaviors and coercive control are usually included in the psychological abuse figures; however, studies that have presented separate results for this form of violence have shown that around 19% of males and 25% of females are victims of these practices (Bonomi et al., 2012). Nevertheless, a recent systematic review of the prevalence of the different forms of teen violence in Europe confirmed that the reported rates vary widely, due in part to sample characteristics and study location but also to the use of different definitions, measures and research designs (Tomaszewska and Schuster, 2021).

### Theories That Explain Teen Dating Violence

Multiple theories have been proposed to explain the use of violence in adolescence and adulthood related to early experiences. Social learning theory has stressed, in general, how the exposure to violence (i.e., advised, witnessed, and being victimized) generates and shapes beliefs and attitudes that support violence through definitions, differential association and reinforcement, and imitation (Akers and Jennings, 2019). Social information processing theory outlines how cognitive processes influence behavioral responses in a child’s interpersonal or social interactions through the cognitive schemas established in their early close relationships (e.g., with caregivers); thus, abused children tend to have hostile attributional bias patterns which increase the likelihood of aggressive behavioral outcomes (Milner, 1993). Similarly, attachment theory posits another psychodynamic notion in which the early bond between the child and their caregiver creates the basis for the child’s internal working model; if the transmission of patterns of relating is poor, this may result in a hostile worldview due to caregiver’s ambivalence, rejection, or abuse, which influences how the child interacts with their interpersonal environment and increases the likelihood of aggressive responses (Zeanah and Zeanah, 1989). Research has provided support for the mechanisms involved in social learning theory, social information processing theory, and attachment theory (Dodge et al., 1990). These theories, along with neuropsychological models and behavioral genetics (Rhee and Waldman, 2002), can be comprehensively framed under the intergenerational transmission of violence – also known as the cycle of violence—to explain the mechanisms through which violence in childhood within the family may lead to the perpetuation of violence in later life (Widom and Wilson, 2015). Meta-analytical evidence from longitudinal studies has confirmed this association (Braga et al., 2018). Applied to dating violence, the current evidence is consistent with the background–situational theory on dating abuse perpetration (Riggs and O’Leary, 1989). This theory posits that the interplay of background or distal factors shapes individuals’ aggressive behavior generally; the exposure to early violence and abusive environment plays a salient role, while situational or proximal factors influence or trigger partner conflict or aggression in specific situations and contexts (Rothman, 2018). This distal link through the long-term impact of childhood adversity on future intimate relationships has been found in the literature (Smith‐Marek et al., 2015). This association has been used to examine violence in partner relationships or the reversal roles of victims and perpetrators across the life course (Haselschwerdt et al., 2019). Existing meta-analyses have not completely elucidated the phenomenon but have documented a small-to-medium effect size linking childhood family violence and school bullying to involvement in dating violence (Emanuels et al., 2022).

### Early Factors That Predict Teen Dating Violence

Previous research has shown that individual and family factors in early stages of life are essential to understanding later aggression in partner relationships. Exposure to dynamics of family violence during childhood may be particularly decisive (Manchikanti Gómez, 2011). Aggressive dynamics imply frequent coercive parenting practices to monitor the child, which developmentally acquaints the child with aggressive or disrupting behaviors (that vary according to individual differences) and potentially lead to conflictive parent-child relationships (Lahey et al., 1999). Thus, two distinct but related pathways that lead to teen dating violence from childhood and early adolescence have been depicted (Makin-Byrd et al., 2013). The first is an aggressive and conflictive family climate that legitimates and shapes future aggressive patterns in children’s interpersonal relationships, which begin in childhood and result in the perpetration of dating violence in late adolescence. This pathway can be described as a distal process in which early experience plays a unique role in later perpetration. Second, because of the violent family dynamic, children reach adolescence with externalizing problems that contribute to negative outcomes, behavioral problems and conflictive relationships at school and establish a pattern of aggressive responses or a propensity to use aggression to achieve one’s objectives. This second pathway can be flagged as a transitional model where school and peers in early adolescence become the main influence for fostering negative behavioral outcomes that may lead to dating violence.

#### Parental monitoring

Youth who are neglected, as characterized by a low level of monitoring and supervision, may be at greater risk of dating violence (Howard et al., 2003). However, although neglectful parenting is clearly an important contributor to aggressive behaviors in both boys and girls, gender seems to moderate the association between this predictor and physical dating violence; parental monitoring is inversely linked to dating violence for boys (Miller et al., 2009), though other studies have found the same relationship for girls (Foshee et al., 2015). In a longitudinal study with US Midwestern adolescents from middle through high school, both male and female perpetrators of physical/threatening behavior started out lower than non-perpetrators in parental monitoring and declined slightly over time. However, for verbal teen dating violence, parental monitoring was important in distinguishing between perpetrators and non-perpetrators among females but not males (Espelage et al., 2020). Another longitudinal study with early adolescent Latino and African American youth found that indicators of social control (mothers’ knowledge of their child’s whereabouts, high strictness, and conservative sexual attitudes) were associated with a lower likelihood of dating victimization especially in the case of Latinos and girls (East and Hokoda, 2015).

#### Child victimization

A review of the literature has suggested that exposure to parenting practices based on the use of corporal punishment, rejection, and coercion increases the risk of future intimate partner violence (Schwartz et al., 2006). Longitudinal studies have found that corporal punishment and childhood abuse predict dating violence involvement. Being hit by an adult with the intention of harm as a child was found to be a significant predictor for being a victim of serious physical dating violence in adolescence in both males and females (Foshee et al., 2004). Through a path model of longitudinal data from the Raising Healthy Children (RHC) project, one study detected that child maltreatment before 13 years of age was a significant predictor of later physical teen dating violence victimization in both girls and boys (Maas et al., 2010). Using data from both waves of the Dating Violence Among Latino Adolescents study, child maltreatment, conventional crime, and polyvictimization (measured at between 12 and 18 years of age) were found to be predictive of dating violence victimization one year later (Cuevas et al., 2020). Similarly, a close relation between peer victimization and dating violence victimization was confirmed in a longitudinal study (Brooks-Russell et al., 2013). Strong associations have also been found between the perpetration of violence against peers and perpetration of dating violence (Foshee et al., 2014), while both the perpetration of violence against peers (Foshee et al., 2004) and reporting being bullied (Adhia et al., 2019) have been found to predict later dating violence victimization. Bullying perpetration had a significantly stronger effect on teen dating violence perpetration in girls than in boys, and victimization only predicted teen dating violence perpetration in boys (Walters and Espelage, 2018). A recent meta-analysis has suggested that bullying and dating violence may be different manifestations of the same underlying violent dispositions (Zych et al., 2021), although more longitudinal studies are needed to confirm these results.

#### Internalizing and externalizing problems

Early childhood externalizing problems, especially behavioral problems, are among the most robust predictors of later partner violence. A prospective longitudinal data set from the Christchurch Health and Development Study was used to assess the influence of the developmental timing of conduct problems on partner violence perpetration and victimization in early adulthood (Woodward et al., 2002). These data indicated that parent and teacher reports of early antisocial behavior (8–12 years old) were associated with higher rates of partnership difficulties at age 21 than adolescent antisocial behavior. Focusing on dating violence, one longitudinal study of children followed from kindergarten entry to the age of 18 years showed that child *aggressive-disruptive behavior* at home at age 6 uniquely predicted later perpetration of dating violence, victimization and total violence exposure (Makin-Byrd et al., 2013). A meta-analysis also found that high-risk behaviors, such as externalizing behaviors, being violent towards peers, risky sexual behaviors, having carried a weapon, and substance use were all significant risk markers for physical teen dating violence victimization (Spencer et al., 2020). No longitudinal studies have analyzed the possible relation between early internalizing problems and teen dating violence perpetration and victimization, but *depression* has been found to be a strong predictor in adolescents (Ulloa et al., 2014), as has *anxiety* (Brooks-Russell et al., 2013).

#### Gender parity

A critical aspect of the lack of consensus in dating violence studies is the gender parity of violence in dating relationships. This question brings together two opposing perspectives, that is, whether the gender patterns of perpetration and victimization are symmetrical or asymmetrical (Eisner, 2021). The ongoing debate presents two divergent positions in the understanding of the ontology of dating violence, the validity of the epistemological approaches, the measurements and ﻿operationalization employed, the results obtained, and their interpretations (Hamby, 2017). The field, therefore, lacks robust findings on the effect of gender on teen dating violence. Studies exploring bidirectional longitudinal associations between victimization and perpetration of dating violence in adolescents are scarce. A recent meta-analysis found that the strongest risk marker for physical teen dating violence victimization was the perpetration of physical dating violence (Spencer et al., 2020).

#### Family structure and parental education

Family structure also seems to be a relevant variable. The correlates of psychological and minor physical dating violence victimization were assessed in a nationally representative sample of adolescents from the National Longitudinal Study of Adolescent Health (Halpern et al., 2001). Living in a non-traditional family structure, i.e., a structure not comprising two biological parents, was found to be a predictor of dating violence victimization for males. Other longitudinal studies have found that family structure is a relevant variable for both sexes in explaining involvement in dating violence (Foshee et al., 2005). Also, a lower level of parental education was positively related to dating violence involvement in the National Longitudinal Study of Adolescent Health (Halpern et al., 2001), and a higher level presented a significant negative association with moderate physical dating violence in another longitudinal study (Foshee et al., 2008).

## Current Study

This study seeks to extend current knowledge by testing the early predictors of teen dating violence. In order to do so, the existence of sub-groups or classes of adolescents characterized by distinct patterns of dating violence involvement is explored. Then, the likelihood of belonging to these classes (or developing these profiles) is determined based on relevant predictors, such as previous experiences of victimization by peers and caregivers, psychological symptoms of emotional distress (such as aggressive behavior, anxiety, and depression), and parental monitoring. The effect of gender, as well as two other relevant variables highlighted by previous longitudinal studies (namely, family structure and parental education), in which non-traditional family structure and parents’ low educational level are positively related with teen dating violence, are also analyzed.

## Methods

### Procedure

Data were drawn from the Zurich Project on the Social Development from Childhood to Adulthood (z-proso), a longitudinal study of a sample of urban adolescents containing extensive multiwave data (Ribeaud et al., 2022). The target population consisted of all 2520 children who entered first grade at one of the 90 public primary schools in the city of Zurich, in 2004. Thus, 56 schools were randomly selected and all children entering first grade this year at one of these schools were invited to participate via their parents, giving a target sample of 1675 children. Out of the target sample, parents of 1366 children (82%) allowed the participation of their child in the surveys and 1240 mothers and fathers (74%) agreed to participate in the surveys themselves. The sample is representative of the target population, with a slight overrepresentation of low socio-economic status (SES) ethnic minorities^1^.

In the first three waves (ages 7 to 9), informed consent was obtained from the parents of participating children and renewed in data collection for wave 4 (when participants were 11 years old). From wave 5 (at age 13) children were able to provide direct informed consent, but their parents retained the right to opt their child out of the study. Informed consent by the youth was renewed at wave 7 (age 17).

Data were collected from children, their parents, and their teachers. Parent interviews at child aged 7, 8, 9 and/or 11 were conducted with the primary caregiver, usually the mother (93.9%), followed by the father (5.2%) and foster or step-parent (0.9%). Interviews were usually carried out at the mother’s home using a computer-aided personal interview (CAPI). Given the highly multicultural population structure the standardized interviews were conducted by specially trained native speakers in nine different languages. The interviews typically took about an hour and participating parents received vouchers worth 20–50$ as participation incentives. The teachers of all participating children in waves 1 to 7 (ages 7 to 17) were invited to complete postal surveys. The instrument consisted of a one-page form related to each participant in the teacher’s class (5–10 min. to complete), plus a questionnaire for the class and the school (5–10 min.). Participation was mandatory for all teachers in the first three waves (ages 7 to 9). After that, teachers who had to complete more than 7 questionnaires were offered book vouchers worth about 50$ as an incentive.

Standardized child interviews (CAPI) were conducted by specially trained interviewers during regular school lessons (45 min.) at ages 7, 8, and 9. The instruments were specially designed for this age group and were mainly game based. Participants received a sticker as a giveaway. From wave 4 (at age 11), the methodology was changed to classroom-based paper-and-pencil questionnaires. The survey sessions were conducted by 2–3 research assistants and lasted for 60–90 minutes, and they took place during regular school lessons in wave 4, but during leisure time from wave 5 onwards (age 13). Youth were incentivized with the equivalent of 60$ in cash.

All data were collected in accordance with the Swiss legislation on data protection and human research. The most recent review by the Ethics Committee of the Faculty of Arts and Social Sciences of the University of Zurich was conducted in early 2018.

### Sample

Data were based on the first (when participants were *M*_*age*_ = 7.45 years old, *SD* = 0.40) and seventh (when participants were *M* = 17.44 years old, *SD* = 0.37) waves of the z-proso, which will henceforth be referred to as T1 and T7. The study sample included only the participants who reported having a dating partner in the previous twelve months at T7. After excluding the participants who did not have a dating partner in the last year and those who had not responded to at least half of the items in the dating violence instrument (*n* = 2), the study sample consisted of 644 adolescents (57.14% female, *M* = 17.47, *SD* = 0.37). Although over 90% of the subjects were born in Switzerland, 60% of the parents were born abroad (from over 90 different countries), so it can be considered a particularly multiethnic sample. At T1, most participants (*n* = 482) lived with both biological parents, 16.14% belonged to a one-biological parent family, 5.60% shared the household with a biological parent and a stepparent and 3.41% lived in other structures (e.g., in residential care or foster families). At T7, 97.8% were still living with one or two of their parents, seven (1.1%) were living in youth centers, residential schools or other types of residence under adult supervision, three (0.5%) were living on their own, three (0.5%) with their life partner and one (0.3%) in a flat sharing community. In 72 cases (11.2%), the parents divorced when their child was between 7 and 18 years old. In almost a quarter of the families (24.3%), at least one of the parents had a university degree when the participating child was 15 years old.

In terms of the characteristics of their romantic relationship, most participants (29.4%) reported having dated their partner for three months or less, 22.8% for 4–6 months, 17.3% for less than a year, 21.7% for 1 to 2 years, 8.5% for 2 to 5 years and 0.5% for 6 years or more. Most reported their relationship to be very good (40.6%) or relatively good (41.2%), 15% reported it to be relatively bad and 3.2% reported it to be very bad. Almost all couples (96.7%) were heterosexual.

### Measures

#### Dating violence

Physical dating violence victimization and perpetration in the 12 months preceding the survey was self-reported by the youth at T7. Participants were asked to refer to their current or most recent relationship, defining an intimate partner as “someone with whom you’ve already been together with for a long time, or someone with whom you were together with for only a short amount of time (but for minimum 1 week), and regardless of whether you had a sexual relationship”. In view of previous work highlighting the need for a broad definition of romantic relationship in adolescents (Furman and Hand, 2006), we did not distinguish couples by duration or by whether they were heterosexual/homosexual. The scale consisted of 28 items, namely, 14 items reported twice—once from the perspective of the victim and once from that of the perpetrator. Respondents had to provide answers on a 4-point Likert scale, ranging from “never” to “more than 9 times” regarding six items on physical violence (i.e., In the last month, has your partner/have you slapped or scratched you/your partner; bitten or kicked you/your partner; pushed, shoved or grabbed you/your partner; hit you/your partner with his/her/your fist or with a hard object; twisted your/your partner’s arm or bent back your/his/her fingers), four on controlling behaviors (i.e., checked your/your partner’s mobile phone, limited contact with your/your partner’s friends, prevented you/your partner from meeting people or asked you/your partner with whom and where you were), and four on sexual aggression or harassment. Due to the low endorsement of the latter category, the four sexual aggression items were excluded from the current analysis. Therefore, 20 items were used, 10 for victimization and 10 for perpetration respectively. Their raw frequencies and percentages are shown in Supplementary Material (Table 1), also divided by gender. A table showing Spearman correlations is included in the same section. Since the disproportional distribution in the endorsement of different choices hampered the data analysis, these variables were converted as follows: participants who responded “Never” to these items were assigned a “0” (no victimization or perpetration), participants who responded “1 to 3 times” or “4 to 9 times” to any of the items were assigned a “1” (considered as occasionally experiencing victimization or perpetrating dating violence) and participants answering “Over 9 times” to any of these items were assigned a “3” (a category indicating frequent victimization or perpetration).

#### Parental monitoring

Monitoring was measured at T1 (i.e., when children were 7 years old) using 10 items that main caregivers had to rate on a four-point Likert-type scale (i.e., from “never” to “often/always”) to describe how they supervised their child (e.g., “You are so busy that you forget where your child is and what he\she is doing.”). Items were based on the Alabama Parenting Questionnaire (APQ, Shelton et al., 1996). The mean of all items was computed. Cronbach’s alpha for this scale was 0.64. Psychometric properties of the instruments have been assessed (Essau et al. (2006)).

#### Corporal punishment

Corporal punishment was assessed at T1 using three items that main caregivers had to rate in terms of frequency on a four-point Likert-type scale (i.e., from “never” to “often/always”) to describe what they would do when the child does something wrong (i.e., whether they spanked them, slapped them and/or hit them with an object). Cronbach’s alpha for this scale was 0.53.

#### Bullying

This variable was converted into a categorical one based on the assessment of teachers at T1 through teachers’ reports and was grouped into three categories as follows: *Incipient perpetrator*. Bullying at age 7 was measured by means of a teacher-reported item on whether “the child dominates others”. Teachers used a 5-point Likert scale ranging from “not at all true” to “very true”. Children whose teacher rated them with a score of 4 or more on these items were considered perpetrators. *Victimization*. Whether the child was bullied by his/her peers at age 7 was measured using the item “child is bullied”. Teachers used a 5-point Likert scale ranging from “not at all true” to “very true”. Children whose teacher rated them with a score of 4 or more on this item were considered victims of bullying. *Bullying overlap*. If a child had a score of 4 (true) or more in both bullying victimization and perpetration, they were considered a case of bullying overlap in which they played both roles (i.e., victim and perpetrator). They were hence excluded from the incipient perpetrator and victim categories and counted as “overlap”. All the information regarding the psychometric properties of this scale can be found in.

#### Aggression

Anger was measured at T1 through self-reports, using an adapted computer-based multimedia 12-item version of Tremblay’s Social Behaviour Questionnaire (SBQ; Tremblay et al., 1991) encompassing physical, reactive, and proactive aggression; and fits the needs of an anonymous assessment of pro-and anti-social behaviors among primary school children. The instrument consists of a series of drawings that display specific behaviors of a child called “Tom” or “Tina”, depending on the child’s gender. For each drawing the child is asked by a voice recorded on the computer whether he/she happens to do what is shown on the drawing (e.g., “Have you ever stolen something in a store?”; “When you’re mad at someone, do you sometimes say bad things behind the other person’s back?”). There are “yes” and “no” buttons at the bottom of each screen and the child is instructed how to use them. Cronbach’s alpha for the overall aggression scale was 0.71.

#### Anxiety and depression

These internalizing ﻿symptoms were also measured at T1 using nine items of the adaptation of SBQ (e.g., “do you have to cry sometimes, like Tom/Tina?”) in a dichotomous form, “yes” or “no”. Cronbach’s alpha for this scale was 0.62.

#### Sociodemographics

Gender, parental level of education and family structure were included in the multinomial logistic regression to check whether the probability of belonging to a latent class differed between boys and girls at age 17. Parental level of education was coded as “1” if at least one of the parents held a university degree and as “0” if otherwise. Family structure was coded as a categorical variable using the following four levels: “two biological parents”, “one biological parent”, “one biological and a stepparent”, “others”). This information was reported by parents at T1.

### Missing Data

Before running the analysis, missing data were carefully considered. The dataset included 644 adolescents that had reported having a dating partner during the last year, with 6.16% of missing data. Missing data per variable ranged from 0.15% to 22.04%, the variables reported by adults (i.e., teachers or parents) being the ones with the most missing data. The pattern of missing data was tested through Little’s Missing Completely at Random (MCAR) Test (Little, 1988). Missing data were found not to be MCAR, so the full information likelihood (FIML) solution was chosen to handle the dataset. This method has been shown to perform efficiently with data missing at random (MAR) or not at random (NMAR) and with amounts of missing data ranging from 20% to 50% (Johansson and Karlsson (2013)). Imputation was done using the mice package (van Buuren, and Groothuis-Oudshoorn, 2011) in R (R Core Team, 2015).

### Data Analysis

In order to classify participants into different categories a latent class analysis was performed (LCA; Collins and Lanza, 2013). This type of analysis has the advantage of classifying subjects according to their response pattern to specific questions, rather than assuming the categories on a theoretical basis. To organize them into categories through this analysis, the participants’ responses to the 20 items on dating violence victimization and perpetration were used as single variables (six physical and four controlling in each case). The software was set to test for 100 different starting values to ensure that the best solution was found (Oberski, 2016). Models with 1 to 10 classes were tested and the one that provided the best fit according to the Akaike Information Criterion (AIC) and Bayesian Information Criterion (BIC) values, where the lowest values indicated the best fit (Collins and Lanza, 2013), but also considering the entropy (Celeux and Soromenho, 1993) and interpretability of the model was selected. The LCA was performed using the poLCA package (Linzer and Lewis, 2011) in R (R Core Team, 2015). The estimated probabilities of class membership to label the classes obtained were used. Then, each subject was assigned to a latent class according to the maximum probability of belonging to that class.

Finally, after testing the preliminary bivariate analyses, a multinomial regression model to predict the outcome variable—dating violence—at age 17 using the predictors and other covariates at age 7 was run. Multicollinearity was checked through variance inflation factors (VIF) assumptions. Unstandardized coefficients were reported, and odds ratios (OR) were calculated for each predictor with their confidence interval (set at 95% CI). Pseudo R squared was obtained to estimate the goodness of fit for each model. Analyses were performed in R (R Core Team, 2015).

## Results

### Coexistence of Teen Dating Violence Perpetration and Victimization

Table 1 presents the different LCA models obtained with their fit indexes. The model with three latent classes based on its low BIC and AIC was chosen, as well as its entropy and interpretability. This was also more parsimonious than the model with the lowest BIC value (with four classes) and had a higher entropy. Using the same criteria, alternative analyses including answers “between four and nine times” in any item as “frequent violence” instead of “occasional” were run, leading to the same model selection and similar results.

**Table 1:** Latent class analysis model for victims and perpetrators of dating violence (*n* = 644)

Number of classes	*Residual Df*	χ^2^ *(p)*	G^2^ *(p)*	BIC	AIC	Entropy^a^
1	620	3836 (0.00)	3797.674 (1.00)	9452.16	9344.936	
2	595	3452 (0.00)	2227.356 (1.00)	8133.535	7914.535	0.83
**3**	**570**	**3252** (**0.00)**	**1831.184** (**1.00)**	**7899.055**	**7568.445**	**0.84**
4	545	1260 (0.00)	1631.195 (1.00)	7860.759	7418.457	0.83
5	520	1269 (0.00)	1508.239 (1.00)	7899.496	7345.501	0.83
6	495	1005 (1.00)	1405.121 (1.00)	7958.07	7292.383	–
7	470	9931 (1.00)	1318.742 (1.00)	8033.384	7256.004	–
8	445	9945 (1.00)	1247.316 (1.00)	8123.650	7234.578	–

Solution in bold is the selected model

*BIC* Bayesian information criterion, *AIC* Akaike information criterion

^a^Entropy could not be computed for models with 6 or more classes due to very low posterior error

**Fig. 1:**
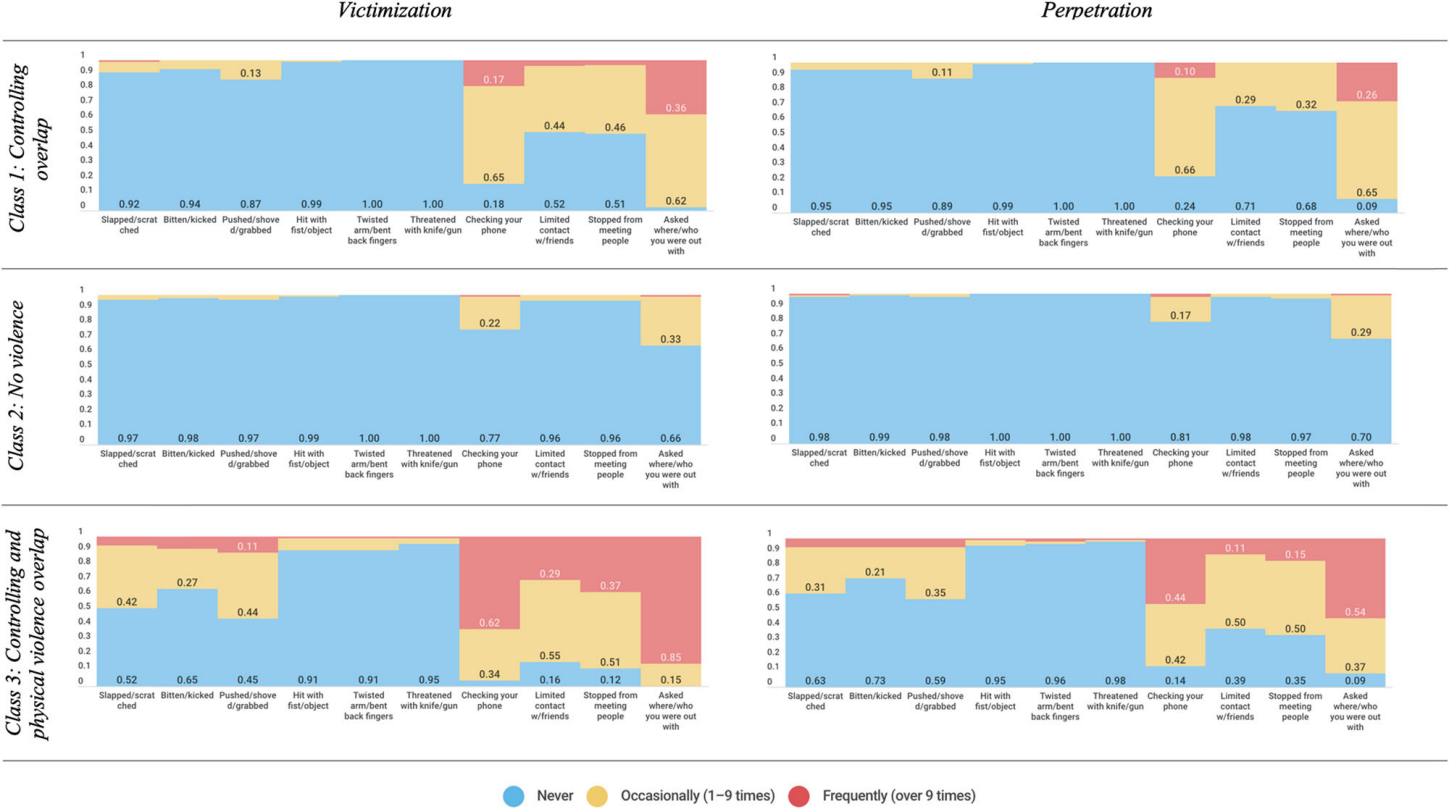
Violent measure endorsement across the latent classes

Table 2 and Fig. 1 show the probability of class membership for each item response. Based on these values, the first class was labeled as “Controlling overlap”, since the subjects in this group were likely to experience both victimization and perpetration of dating violence, but particularly in the items referring to controlling behaviors (e.g., checking your partner’s phone, or your partner checking yours). The second class was labeled as “No violence” since the participants included in this group hardly ever perpetrated or experienced physical or controlling dating violence. Finally, the third class was labeled as “Controlling and physical violence overlap” because participants with the highest probabilities of belonging to this group experienced and perpetrated both physical and controlling dating violence.

**Table 2:** Latent class variable probabilities for a three-class model

	Class 1: Controlling overlap		Class 2: No violence		Class 3: Controlling and physical violence overlap	
Predicted class membership (SE^a^)	0.45 (0.02)		0.42 (0.02)		0.13 (0.02)	
Probability of class membership (SE^a^)						
	Victimization	Perpetration	Victimization	Perpetration	Victimization	Perpetration
Physical violence						
Slapped/scratched						
Never	0.92 (0.01)	0.95 (0.02)	0.97 (0.48)	0.98 (0.01)	0.52 (0.08)	0.63 (0.08)
Occasionally (1–9 times)	0.07 (0.02)	0.05 (0.02)	0.03 (0.01)	0.01 (0.00)	0.42 (0.08)	0.31 (0.08)
Frequently (over 9 times)	0.01 (0.01)	0.00 (0.00)	0.00 (0.49)	0.01 (0.00)	0.06 (0.05)	0.06 (0.00)
Bitten/kicked						
Never	0.94 (0.02)	0.95 (0.02)	0.98 (0.24)	0.99 (0.24)	0.65 (0.12)	0.73 (0.10)
Occasionally (1–9 times)	0.06 (0.02)	0.05 (0.02)	0.02 (0.02)	0.01 (0.01)	0.27 (10)	0.21 (0.09)
Frequently (over 9 times)	0.00 (0.00)	0.00 (0.00)	0.00 (0.24)	0.00 (0.24)	0.08 (0.09)	0.06 (0.05)
Pushed/shoved/grabbed						
Never	0.87 (0.03)	0.89 (0.03)	0.97 (0.02)	0.98 (0.01)	0.45 (0.12)	0.59 (0.10)
Occasionally (1–9 times)	0.13 (0.03)	0.11 (0.03)	0.03 (0.02)	0.02 (0.01)	0.44 (0.10)	0.35 (0.10)
Frequently (over 9 times)	0.00 (0.00)	0.00 (0.00)	0.00 (0.00)	0.00 (0.00)	0.11 (0.12)	0.06 (0.05)
Hit with fist/object						
Never	0.99 (0.00)	0.99 (0.00)	0.99 (0.00)	1.00 (0.00)	0.91 (0.12)	0.95 (0.04)
Occasionally (1–9 times)	0.01 (0.00)	0.01 (0.00)	0.01 (0.00)	0.00 (0.00)	0.08 (0.06)	0.04 (0.04)
Frequently (over 9 times)	0.00 (0.00)	0.00 (0.00)	0.00 (0.00)	0.00 (0.00)	0.01 (0.00)	0.01 (0.02)
Twisted arm/bent back fingers						
Never	1.00 (0.00)	1.00 (0.00)	1.00 (0.00)	1.00 (0.00)	0.91 (0.06)	0.96 (0.04)
Occasionally (1–9 times)	0.00 (0.00)	0.00 (0.00)	0.00 (0.00)	0.00 (0.00)	0.08 (0.06)	0.02 (0.03)
Frequently (over 9 times)	0.00 (0.00)	0.00 (0.00)	0.00 (0.00)	0.00 (0.00)	0.01 (0.00)	0.02 (0.03)
Threatened with knife/gun^b^						
Never	1.00 (0.00)	1.00 (0.00)	1.00 (0.00)	1.00 (0.00)	0.95 (0.05)	0.98 (0.04)
Occasionally (1–9 times)	0.00 (0.00)	0.00 (0.00)	0.00 (0.00)	0.00 (0.00)	0.04 (0.05)	0.01 (0.02)
Frequently (over 9 times)	0.00 (0.00)	0.00 (0.00)	0.00 (0.00)	0.00 (0.00)	0.01 (0.02)	0.01 (0.04)
Controlling behaviors						
Checking your phone						
Never	0.18 (0.03)	0.24 (0.03)	0.77 (0.03)	0.81 (0.03)	0.04 (0.03)	0.14 (0.07)
Occasionally (1–9 times)	0.65 (0.03)	0.66 (0.03)	0.22 (0.03)	0.17 (0.03)	0.34 (0.09)	0.42 (0.07)
Frequently (over 9 times)	0.17 (0.03)	0.10 (0.02)	0.01 (0.00)	0.02 (0.01)	0.62 (0.10)	0.44 (0.08)
Limited contact w/friends						
Never	0.51 (0.04)	0.71 (0.03)	0.96 (0.01)	0.98 (0.01)	0.16 (0.07)	0.39 (0.11)
Occasionally (1–9 times)	0.44 (0.04)	0.29 (0.03)	0.04 (0.01)	0.02 (0.01)	0.55 (0.09)	0.50 (0.10)
Frequently (over 9 times)	0.04 (0.02)	0.00 (0.00)	0.00 (0.00)	0.00 (0.00)	0.29 (0.08)	0.11 (06)
Stopped from meeting people						
Never	0.51 (0.04)	0.68 (0.03)	0.96 (0.02)	0.97 (0.01)	0.12 (0.06)	0.35 (0.11)
Occasionally (1–9 times)	0.46 (0.04)	0.32 (0.03)	0.04 (0.02)	0.03 (0.01)	0.51 (0.09)	0.50 (0.10)
Frequently (over 9 times)	0.03 (0.01)	0.00 (0.00)	0.00 (0.00)	0.00 (0.00)	0.37 (0.09)	0.15 (0.07)
Asked where/who you were out with						
Never	0.02 (0.01)	0.09 (0.02)	0.66 (0.04)	0.70 (0.04)	0.00 (0.00)	0.09 (0.07)
Occasionally (1–9 times)	0.62 (0.04)	0.65 (0.04)	0.33 (0.04)	0.29 (0.04)	0.15 (0.06)	0.37 (0.08)
Frequently (over 9 times)	0.36 (0.04)	0.26 (0.07)	0.01 (0.00)	0.01 (00)	0.85 (0.06)	0.54 (0.08)

^a^Standard error

^b^No participant answered “Over 9 times” to this item

The most prevalent class was the “controlling behavior overlap” class, suggesting that it is very common among young couples to control each other’s phone, limit their contacts and stop them from meeting people. Regarding distribution by gender, 38.8% of boys and 49.7% of girls were classified as belonging to the “Controlling behavior overlap” group, 48.9% of boys and 37.5% of girls to the “No violence” group, and 12.3% of boys and 12.8% of girls to the “Controlling and physical violence overlap” group.

### Victim-Perpetrator Group Predictors

Table 3 presents the results of the multinomial regression model to predict the category to which each subject belonged by means of the selected predictors and covariates.

**Table 3:** Multinomial logistic regression to predict latent class membership

	*β* (SE)	OR (CI 95%)
*Outcome reference class* *=* *“No violence”*		
**Controlling overlap**		
* Intercept*	−0.47 (0.25)	1.23 (0.38–1.03)
**Predictors**		
Corporal punishment at age 7	0.23 (0.20)	1.26 (0.85–1.87)
Victim of bullying at age 7	0.93 (0.75)	2.54 (0.58–11.01)
Incipient perpetrator of bullying at age 7	0.38 (0.36)	1.47 (0.72–3.00)
Overlap bullying at age 7	2.04 (1.12)	7.70 (0.85–69.69)
Aggression at age 7	−0.19 (0.59)	0.83 (0.26–2.63)
Anxiety and depression at age 7	0.33 (0.43)	1.40 (0.59–3.19)
Parental monitoring	0.23 (0.28)	1.25 (0.73–2.15)
**Covariables**		
*Gender (reference* = *Male)*		
Female	0.48 (0.19)**	1.62 (1.13–2.33)
*Family structure (reference* = *both biological parents)*		
One biological parent	0.11 (0.24)	1.12 (0.70–1.78)
One biological parent and a stepparent	0.86 (0.43)*	2.37 (1.02–5.48)
**Other**	−0.10 (0.59)	0.90 (0.57–2.88)
*Parental level of education (reference* *=* *lower than university)*		
University degree or higher	−0.56 (0.21)**	0.57 (0.38–0.87)
**Controlling and physical violence overlap**		
*Intercept*	−1.87 (0.39)*	0.15 (0.07–0.33)
**Predictors**		
Corporal punishment at age 7	0.67 (0.28)*	1.95 (1.12–3.40)
Victim of bullying at age 7	2.23 (0.78)**	9.71 (2.10–44.89)
Incipient perpetrator of bullying at age 7	0.76 (0.49)	2.15 (0.83–5.57)
Overlap bullying at age 7	1.47 (1.46)	4.36 (0.25–76.77)
Aggression at age 7	−0.37 (0.90)	0.69 (0.12–4.08)
Anxiety and depression at age 7	0.53 (0.65)	1.70 (0.47–6.16)
Parental monitoring	0.04 (0.41)	1.04 (0.46–2.33)
**Covariables**		
*Gender (reference* = *Male)*		
Female	0.27 (0.29)	1.31 (0.75–2.29)
*Family structure (reference* = *both biological parents)*		
One biological parent	−0.26 (0.24)	0.77 (35–1.68)
One biological parent and a stepparent	1.30 (0.56)*	3.68 (1.24–10.94)
***Other***	0.68 (0.73)	1.96 (0.49–8.75)
*Parental level of education (reference* *=* *lower than university)*		
University degree or higher	−1.00 (0.38)**	0.37 (0.17–0.77)

Pseudo *R*^2^ obtained for the model was 0.05 (McFadden, 1974), 0.09 (Cox and Snell, 1989), 0.10 (Nagelkerke, 1991)

*p < 0.05; **p < 0.01; ***p < 0.001

Gender, family structure and parents’ level of education were the only significant predictors for belonging to the “controlling behavior overlap” dating violence category. According to the results, girls were 1.61 times more likely to belong to this category than boys, and adolescents from a family with a stepparent were almost 2.5 times more likely to belong to this category than their counterparts. Participants that had at least one parent with a completed bachelor’s degree or higher were significantly less likely to belong to this category than adolescents whose parents had a high school degree or lower.

Having suffered corporal punishment and bullying at the age of 7 significantly increased the likelihood of belonging to the “controlling and physical violence overlap” dating violence category. Children who had experienced corporal punishment by the age of 7 were almost twice more likely to both perform and suffer physical and controlling dating violence at the age of 17. Children who were bullied at the age of 7 were around 10 times more likely to belong to this category, although the confidence interval was quite broad. In terms of demographic characteristics, belonging to a family with a stepparent increased the likelihood of belonging to this category more than three-fold. Having at least one parent with a university degree significantly decreased the likelihood of belonging to the “controlling and physical violence overlap” dating violence class.

## Discussion

The earliest predictors of teen dating violence remain relatively uncharted. The need for early identification of those at risk of committing or experiencing dating violence at a later stage in their lives, in order to be able to implement effective prevention measures, has been largely ignored. Therefore, the main aim of the present study was to address this important gap in the literature and to present an analysis of childhood individual, family and peer risk factors for teen dating violence from a longitudinal perspective that may help predict perpetration and victimization 10 years later. The study addresses different patterns of perpetration and victimization in teen romantic relationships and also examines differences by gender. Most of the overall associations expected between early childhood risk factors and involvement in teen dating violence were found, with the notable exceptions of aggression and parental monitoring, which were not significantly correlated with the perpetration and victimization of teen dating violence in either boys or girls.

### Coexistence of Teen Dating Violence Perpetration and Victimization

The use of an empirical approach to define roles in teen dating violence is based on a person-centered perspective that allows for the clustering of individuals into groups in a way that minimizes differences within groups and maximizes differences between groups (Swartout et al., 2011). The same methodology used to assign participants from the same sample to bullying roles (Zych et al., 2020) was followed, instead of the a priori classifications of participants. A recent longitudinal study also used this approach, although only to analyze predictors of dating violence perpetration (Saint-Eloi Cadely et al., 2020).

Another strength of the present study is the concurrent investigation of controlling behaviors and physical dating violence. Previous person-centered studies have limited their focus either to psychological (Orpinas et al., 2012) or to physical violence (Orpinas et al., 2013). Studying both forms of teen dating violence together can highlight the similarities in their patterns of perpetration and victimization.

Thus, while some adolescents neither use nor receive violence in their dating relationships, the results show that both girls and boys often engage in mutual controlling behavior and physical aggression in early romantic relationships. Although the reported prevalence rates of teen dating violence vary widely, due to the characteristics of the samples in different studies, the measure of the violence used, the time frame evaluated, and the study location (Tomaszewska and Schuster, 2021), the rates found in the present study for controlling behaviors are higher than previously found (Bonomi et al., 2012). The results show that being involved in controlling and coercive behaviors (for example, “checking your partner’s phone” or “having your phone checked by your partner”) is especially frequent in adolescent dating relationships. In this context, it has been stated that coercive control behaviors are often considered acceptable and desirable expressions of love and concern in adolescents (Bishop and Bettinson, 2018). Such behaviors, like the attitudes that legitimize physical violence, are associated with traditional gender roles, stereotypes and cognitive biases conforming to beliefs about role-related behaviors (Reyes et al., 2016). In fact, adolescents who reported dating violence perpetration were more likely than non-violent ones to report jealousy, verbal conflicts, cheating, or no identity support, but at the same time did not differ in their levels of love and caring in their dating relationships (Giordano et al., 2010), a finding that stresses the importance of relationship contexts and the nuances of power imbalance.

Although several studies have highlighted the mutual nature of teen dating violence (Richards and Branch, 2012), the latent classes that emerged did not suggest the existence of only or mostly adolescent victims or adolescent perpetrators, but a general overlap between these roles. Similarly, another study placed over 90% of participants in a concordant category of psychological dating violence perpetration and victimization (Orpinas et al., 2012).

### Victim-Perpetrator Group Predictors

The rates of teen dating violence perpetration and victimization vary with gender, with girls reporting more controlling behaviors, both as victims and as perpetrators. Similar results have been obtained in previous studies analyzing verbal-emotional abuse in teen dating (Hokoda et al., 2012), including controlling behaviors (Bonomi et al., 2012). Some longitudinal studies have also found that girls tend to perpetrate equal or even more levels of psychological and physical violence than boys (Fernández-González et al., 2020). However, systematic bias in self-reports is a major concern. The possible social desirability bias in boys may lead to the underreporting of physical violence. Similarly, the lower rates of sexual violence reported by both genders may also be related to this type of bias (Wincentak et al., 2017). In addition, some studies have pointed out that girls may over-report dating aggression because of the lower degree of societal sanctions or because they consider their own behavior as having less impact (Foshee, 1996). Whatever the reason, overcoming methodological limitations and refining the instruments used are both crucial.

The association between childhood corporal punishment and being a victim and perpetrator of controlling and physical dating violence has been reported elsewhere. Several longitudinal studies have found that adolescents who have been victims of family abuse present a higher risk of becoming victims or perpetrators of dating violence (Cascardi, 2016). The fact that corporal punishment was assessed at a very early age, and that its outcomes were evaluated 10 years later, adds to the growing literature demonstrating deleterious outcomes associated with corporal punishment.

Furthermore, being bullied at age 7 predicted involvement in controlling and physical dating violence 10 years later. The results confirm the close relation between peer victimization and dating violence, as found in previous longitudinal studies (Foshee et al., 2014). Compared to youth involved in other patterns of violence, youth involved in peer and dating violence as perpetrators and victims are at a greater risk of negative sequelae (Reyes et al., 2018). This should alert parents and educators to the need to intervene in bullying situations as soon as possible, given its long-lasting effects and the perpetuation of victim and perpetrator roles. However, no gender differences were found, in line with recent longitudinal studies (Adhia et al., 2019) but in contrast to other studies analyzing bullying as a predictor of teen dating violence (Walters and Espelage, 2018). Regardless of gender, prior victimization has been consistently related to later victimization. According to some studies, there may be a general propensity for victimization to underlie both prior and later victimization, or, alternatively, prior victimization may directly exacerbate the risk of later victimization by instigating the use of maladaptive strategies to cope with victimization experiences, which in turn increases the risk of repeat victimization (Averdijk et al., 2019). This underlines again the importance of early prevention programs to reduce victimization over the lifespan.

In relation to family structure, adolescents with a family with a stepparent were twice as likely to be victims of controlling behavior and to control the behavior of their dating partner than adolescents belonging to a family with both biological parents living together. It is interesting that this effect was higher than the effect of having another type of family structure, which included foster care. The relation between family structure and teen dating violence has not been analyzed in depth, and very little work has examined the impact of parenting processes on dating violence behaviors. Adolescents living in single-parent households have been found to perpetrate more dating abuse than those living with both parents, and increased involvement in dating abuse perpetration is found to persist throughout adolescence (Foshee et al., 2009). Similarly, living in a non-traditional family structure was found to be a predictor of dating violence perpetration in males (Halpern et al., 2001). The fact that the present study analyzed both victimization and perpetration at the same time adds new evidence of the effect of family structure on teen dating violence. Nonetheless, the underlying factors that could explain these differences in family structures such as family cohesion, stability, or socioeconomic status still need to be explored (Stith et al., 2009).

The negative effect of low parental level of education on teen dating violence behaviors has been reported elsewhere. For example, having a mother with a low level of education was a risk factor for becoming a victim of sexual dating violence (Foshee et al., 2004). Similarly, low parental education was associated with the perpetration of psychological abuse in dating relationships (Foshee et al., 2009). However, the protective effect of a high level of parental education was only significant for girls’ victimization (Foshee et al., 2015), and for dating violence perpetration (see the meta-analysis by Park and Kim, 2018). These conflicting results may reflect the fact that the analysis focused on one dating violence role (victim or perpetrator) and did not take into account the victim-perpetrator overlap in teen dating violence.

### Limitations

Although this study provided relevant findings, certain limitations should be borne in mind when interpreting the results. First, the measures of corporal punishment, parental monitoring and anxiety and depression provided values of reliability that did not reach acceptance. Even though these variables may not fit with the traditional concept of reliability, as discussed in several publications (Lorber and Smith Slep, 2018)) it is important to consider this point when interpreting the results. It has been found that adolescents view behaviors as abusive only in specific contexts, and the fact that the present study did not control for the situations in which the behavior took place may be seen as a limitation (Sears et al., 2006). Another limitation is the fact the gender of the dating partners was not controlled for, which may have influenced the results as reported in studies of college students (Snyder et al., 2018); nor were gender roles and related beliefs examined. It has also been reported that boys appear to underreport the perpetration of dating violence (Hilton et al., 2000). In the present study, the magnitude of potential gender differences in recall biases, misreporting, or willingness to disclose information regarding dating violence was unknown. In addition, the fact that data were collected from a community sample of adolescents might be regarded as a limitation, since in a higher risk sample the results might be different. It would be interesting to try to replicate these findings in higher risk samples. Another important aspect to bear in mind when interpreting the results is sample attrition. Overall, 77% of the initial target sample in T1 participated in T7, the level of attrition being higher in participants coming from a migrant background and with lower levels of education (Ribeaud et al., 2022). An additional limitation is the fact that the study did not examine ethnicity as a potential moderator of predictive associations, even though cultural minorities have been found to present higher rates of dating violence (Wincentak et al., 2017). In addition, within the same race, youth and families differ in important variables such as family structure, and these other variables may contribute to the intragroup variability and explain the predictive associations (Foshee et al., 2005). The use of a longitudinal approach also entails the problem of attrition. In our case, this was quite high since 77% of participants recruited in T1 were still participating in T7 (Ribeaud et al., 2022). However, it should be borne in mind that attrition was higher among participants coming from a migrant background and with low levels of education. Finally, even though the multi-informant approach to the evaluation of children’s emotional and behavioral problems is widely recognized, only low to moderate agreement between informants has been found (De Los Reyes et al., 2015) and the effect of family characteristics and relational aspects on parent/child agreement are important variables that need to be considered (Van Roy et al., 2010). Similarly, other studies extended this previous literature indicating that the lack of congruence between parents’ and children’s reports about parenting behavior is also common (Korelitz and Garber, 2016). Nevertheless, the present study used different informants for different variables, and this may have had an effect on the results obtained that cannot be overlooked. Neither parental, nor teacher’s, nor self-report data can substitute each other in psychosocial assessment (Achenbach et al., 1987).

### Practice Implications

The findings of this study have important implications for clinical practice, policy, and research. Due to its high prevalence and adverse health outcomes (Exner-Cortens et al., 2013), there has been an increased interest in understanding the etiology of adolescent dating violence (Vagi et al., 2013), and in developing more early intervention and prevention programs. However, the fact that adolescent dating violence prevention programs offer a single universal content to all cases ignores the complexity of the problem (Shorey et al., 2012). Addressing teen dating violence by typology or classes would greatly facilitate the development of better and more targeted preventive interventions (Reidy et al., 2016), as has been found in relation to bullying (see Gaffney et al., 2018). In addition, dating violence victimization and perpetration should not be thought of as two independent concepts, since they overlap in important ways. This overlap should be borne in mind in public policies addressed to prevent teen dating violence, since considering them separately can only offer a partial description of the problem, which may influence the effectiveness of the interventions conducted to prevent it (Park and Kim, 2019). Treatment providers and other professionals in contact with adolescents need to be educated on the overlap that exists between victimization and perpetration in order to be able to offer appropriate services (Tillyer and Wright, 2014). Furthermore, gender conceptualizations of victims and perpetrators in teen dating violence need to align with current empirical research (Eisner, 2021). The use of gender-neutral terms for teen dating violence interventions and prevention responses will make it possible to address the needs of all victims and perpetrators. The results indicate that both peers and family should be part of the development of efficient prevention options (Hébert et al., 2019), and that the long-lasting influence of very early experiences in the family and with peers should be taken into consideration.

## Conclusion

The absence of longitudinal studies of early predictors of teen dating violence has limited our understanding of the etiology of adolescent dating violence and the development of early intervention and prevention programs. The aim of the present study was to advance on previous research by shedding light on the early predictors of teen dating violence from a longitudinal perspective. The results supported the idea that violence experienced in middle childhood by peers and caregivers is associated with dating violence victimization and perpetration over a decade later. The experience of violence at early developmental stages puts children at risk of internalizing its use in their teen dating relationships and may assume both roles, victims and perpetrators. Discouraging caregivers from using corporal punishment and focusing on decreasing bullying at elementary school may help lower the prevalence of teen dating violence in adolescence. Future research would also benefit from analyzing the family structure and family education in other samples with different cultural origins and examining gender roles, which would lead us to a more comprehensive understanding of the factors that contribute to teen dating violence.

## Supplementary Information

Supplementary Information

Supplementary Information

## Footnotes

1 Full reports in “https://www.jacobscenter.uzh.ch/en/research/zproso/aboutus/inst_erheb.html”
