# Supplementary material for: Early Childhood Predictors of Teen Dating Violence Involvement at Age 17
Source: J Youth Adolesc. 2022 Aug 6;51(11):2219–34. doi: 10.1007/s10964-022-01664-8 (PMC9508003; doi:10.1007/s10964-022-01664-8)
Supplement: Supplementary file 1 — Supplementary Information [file 10964_2022_1664_MOESM1_ESM.docx]

Table 1

*Item frequencies and percentages for the complete sample, girls and boys*

|  | Overall  (*n* = 644) | | | | Girls  (*n* = 368) | | | | Boys  (*n* = 276) | | | | NA |
| --- | --- | --- | --- | --- | --- | --- | --- | --- | --- | --- | --- | --- | --- |
| In the last month, has your partner | 10 | 1-3 | 4-9 | >9 | 0 | 1-3 | 4-9 | >9 | 0 | 1-3 | 4-9 | >9 |  |
| 1 slapped or scratched you? | 569  (88.5) | 57  (8.8) | 8  (1.2) | 9  (1.4) | 344  (93.5) | 20  (5.4) | 2  (0.5) | 2  (0.5) | 225  (81.8) | 37  (13.5) | 6  (2.2) | 7  (2.5) | 1 |
| 2 bitten or kicked you? | 590  (91.8) | 41  (6.4) | 4  (0.6) | 8  (1.2) | 342  (92.9) | 32  (6.5) | 0  (0.0) | 2  (0.5) | 248  (90.2) | 15  (6.2) | 4  (1.5) | 6  (2.2) | 1 |
| 3 pushed, grabbed or shoved you? | 551  (86.0) | 65  (10.1) | 14  (2.2) | 11  (1.7) | 308  (84.2) | 44  (12.0) | 9  (2.5) | 5  (1.4) | 243  (88.4) | 21  (7.6) | 5  (1.8) | 6  (2.2) | 3 |
| 4 hit you with a fist? | 630  (98.0) | 11  (1.7) | 0  (0) | 2  (0.3) | 362  (98.4) | 5  (1.4) | 0  (0.0) | 1  (0.3) | 268  (97.5) | 6  (2.2) | 0  (0.0) | 1  (0.4) | 1 |
| 5 twisted your arm or finger? | 634  (98.6) | 8  (1.2) | 0  (0) | 1  (0.2) | 364  (98.9) | 3  (0.8) | 0  (0.0) | 1  (0.3) | 270  (98.2) | 5  (1.8) | 0  (0.0) | 0  (0.0) | 1 |
| 6 threatened you with a weapon? | 638  (99.4) | 3  (0.5) | 1  (0.2) | 0  (0.0) | 367  (99.7) | 1  (0.3) | 0  (0.0) | 0  (0.0) | 271  (98.9) | 2  (0.7) | 1  (0.4) | 0  (0.0) | 2 |
| 7 checked your cell phone? | 260  (40.5) | 196 (30.5) | 83  (12.9) | 103 (16.0) | 64  (17.4) | 44  (12.0) | 115  (31.3) | 144  (39.2) | 116  (42.2) | 81  (29.5) | 39  (14.2) | 39  (14.2) | 2 |
| 8 limited your contact? | 420  (65.8) | 141  (22.1) | 43  (6.7) | 34  (5.3) | 220  (60.3) | 92  (25.2) | 29  (7.9) | 24  (6.5) | 200  (73.3) | 49  (17.9) | 14  (5.1) | 10  (3.7) | 6 |
| 9 prevented you from meeting people? | 418  (65.1) | 144 (22.4) | 40  (6.2) | 40  (6.2) | 233  (63.3) | 90  (24.5) | 19  (5.2) | 26  (7.1) | 185  (67.5) | 54  (19.7) | 21  (7.7) | 14  (5.1) | 2 |
| 10 asked about your whereabouts? | 182  (28.4) | 178 (27.8) | 102  (15.9) | 179 (27.9) | 87  (23.6) | 106  (28.9) | 63  (17.2) | 111  (30.2) | 95  (34.7) | 72  (26.3) | 39  (14.2) | 68  (24.8) | 3 |
| In the last month, have you… |  |  |  |  |  |  |  |  |  |  |  |  |  |
| 11 slapped or scratched your partner? | 594  (92.5) | 34  (5.3) | 7  (1.1) | 7  (1.1) | 335  (91.0) | 22  (6.0) | 7  (1.9) | 4  (1.1) | 259  (94.5) | 12  (4.3) | 0  (0.0) | 3  (1.1) | 2 |
| 12 bitten or kicked your partner? | 603 (93.68) | 30  (4.7) | 4  (0.6) | 6  (0.9) | 340  (92.4) | 22  (6.0) | 3  (0.8) | 3  (0.8) | 263  (95.6) | 8  (2.9) | 1  (0.4) | 3  (1.1) | 1 |
| 13 pushed, grabbed or shoved your partner? | 572  (89.0) | 60  (9.3) | 5  (0.8) | 6  (0.9) | 319  (86.9) | 40  (10.9) | 5  (1.4) | 3  (0.8) | 253  (91.7) | 20  (7.2) | 0  (0.0) | 3  (1.1) | 1 |
| 14 hit your partner with a fist? | 626  (99.1) | 5  (0.8) | 0  (0.0) | 1  (0.2) | 365  (99.2) | 2  (0.5) | 0  (0.0) | 1  (0.3) | 271  (98.2) | 3  (1.1) | 0  (0.0) | 0  (0.0) | 2 |
| 15 twisted the arm or finger of your partner? | 638  (99.4) | 2  (0.3) | 2  (0.3) | 0  (0.0) | 367  (99.7) | 0  (0.0) | 1  (0.3) | 0  (0.0) | 271  (98.9) | 2  (0.7) | 1  (0.4) | 0  (0.0) | 2 |
| 16 threatened your partner with a weapon? | 642  (99.7) | 1  (0.2) | 1  (0.2) | 0  (0.0) | 368  (100) | 0  (0.0) | 0  (0.0) | 0  (0.0) | 274  (99.3) | 1  (0.4) | 1  (0.4) | 0  (0.0) | 0 |
| 17 checked the mobile of your partner? | 298  (46.3) | 201 (31.3) | 74  (11.5) | 70  (10.9) | 157  (42.7) | 120  (32.6) | 45  (12.2) | 46  (12.5) | 141  (51.3) | 81  (29.5) | 29  (10.5) | 24  (8.7) | 1 |
| 18 limited contact of your partner? | 499  (77.7) | 104 (16.2) | 29  (4.5) | 10  (1.6) | 276  (75.4) | 64  (17.5) | 21  (5.7) | 5  (1.4) | 223  (80.8) | 40  (14.5) | 8  (2.9) | 5  (1.8) | 2 |
| 19 Have you prevented your partner from meeting people? | 483  (75.6) | 112 (17.5) | 31  (4.9) | 13  (2.0) | 273  (75.0) | 65  (17.9) | 19  (5.2) | 7  (1.9) | 210  (76.4) | 47  (17.1) | 12  (4.4) | 6  (2.2) | 5 |
| 20 Have you asked your partner about his/her whereabouts? | 223  (34.7) | 181 (28.1) | 115  (17.9) | 124  (19.3) | 112  (30.5) | 108  (29.4) | 65  (17.7) | 82  (22.3) | 111  (40.2) | 73  (26.4) | 50  (18.1) | 42  (15.2) | 1 |
