# Supplementary material for: Early Childhood Predictors of Teen Dating Violence Involvement at Age 17
Source: J Youth Adolesc. 2022 Aug 6;51(11):2219–34. doi: 10.1007/s10964-022-01664-8 (PMC9508003; doi:10.1007/s10964-022-01664-8)
Supplement: Supplementary file 2 — Supplementary Information [file 10964_2022_1664_MOESM2_ESM.docx]

Table 2

*Spearman correlations between teen dating violence items*

|  | VP1 | VP2 | VP3 | VP4 | VP5 | VP6 | VC1 | VC2 | VC3 | VC4 | PP1 | PP2 | PP3 | PP4 | PP5 | PP6 | PC1 | PC2 | PC3 |
| --- | --- | --- | --- | --- | --- | --- | --- | --- | --- | --- | --- | --- | --- | --- | --- | --- | --- | --- | --- |
| VP2 | .44 |  |  |  |  |  |  |  |  |  |  |  |  |  |  |  |  |  |  |
| VP3 | .38 | .47 |  |  |  |  |  |  |  |  |  |  |  |  |  |  |  |  |  |
| VP4 | .21 | .28 | .24 |  |  |  |  |  |  |  |  |  |  |  |  |  |  |  |  |
| VP5 | .26 | .31 | .27 | .36 |  |  |  |  |  |  |  |  |  |  |  |  |  |  |  |
| VP6 | .23 | .20 | .20 | .27 | .66 |  |  |  |  |  |  |  |  |  |  |  |  |  |  |
| VC1 | .26 | .17 | .29 | .06^*^ | .11 | .07^*^ |  |  |  |  |  |  |  |  |  |  |  |  |  |
| VC2 | .21 | .17 | .26 | .13 | .15 | .09 | .49 |  |  |  |  |  |  |  |  |  |  |  |  |
| VC3 | .22 | .18 | .23 | .09 | .16 | .12 | .47 | .66 |  |  |  |  |  |  |  |  |  |  |  |
| VC4 | .22 | .19 | .37 | .09 | .11 | .10 | .58 | .53 | .54 |  |  |  |  |  |  |  |  |  |  |
| PP1 | .39 | .20 | .23 | .09 | .07^*^ | -.02^*^ | .19 | .16 | .18 | .19 |  |  |  |  |  |  |  |  |  |
| PP2 | .27 | .56 | .35 | .06^*^ | .19 | .14 | .13 | .12 | .12 | .15 | .38 |  |  |  |  |  |  |  |  |
| PP3 | .28 | .35 | .61 | .09 | .26 | .22 | .18 | .15 | .19 | .21 | .31 | .42 |  |  |  |  |  |  |  |
| PP4 | .07^*^ | .15 | .15 | .22 | .26 | .40 | .11 | .11 | .10 | .09 | .05^*^ | .18 | .13 |  |  |  |  |  |  |
| PP5 | .11 | .19 | .14 | -.01^*^ | .33 | .50 | .11 | .08 | .14 | .10 | -.02^*^ | .22 | .17 | .40 |  |  |  |  |  |
| PP6 | .17 | .09 | .13 | -.01^*^ | .47 | .71 | .07^*^ | .03^*^ | .08 | .07^*^ | -.01^*^ | .21 | .16 | .58 | .71 |  |  |  |  |
| PC1 | .17 | .07^*^ | .17 | -02^*^ | .05^*^ | .05^*^ | .68 | .35 | .33 | .48 | .18 | .15 | .19 | .11 | .10 | .08 |  |  |  |
| PC2 | .16 | .12 | .18 | .03^*^ | .04^*^ | .06^*^ | .34 | .44 | .39 | .36 | .14 | .12 | .15 | .15 | .16 | .11 | .42 |  |  |
| PC3 | .17 | .09^*^ | .20 | -.00^*^ | .03^*^ | .05^*^ | .36 | .32 | .43 | .36 | .15 | .17 | .14 | .10 | .16 | .10 | .42 | .56 |  |
| PC4 | .12 | .07^*^ | .16 | -.01^*^ | -.06^*^ | -.07^*^ | .45 | .35 | .36 | .72 | .17 | .08 | .15 | .02^*^ | -.00^*^ | -.06^*^ | .54 | .41 | .44 |

Note: *n* = 644. ^*^ non-significant at the 5% level.

VP = victim of physical violence, PP = perpetrator of physical violence, VC = victim of control violence, PC = perpetrator of control violence. VP & PP items are: 1. Slap or scratch; 2. Bite or kick; 3. Push, grab or shove; 4. Hit with fist; 5. Twist arm or finger; 6. Threaten with weapon; VC and PC items are: 1. Check mobile; 2. Limit contact; 3. Prevent from meeting people; 4. Ask whereabouts.
